# Potentiation of Thrombin Generation in Hemophilia A Plasma by Coagulation Factor VIII and Characterization of Antibody-Specific Inhibition

**DOI:** 10.1371/journal.pone.0048172

**Published:** 2012-10-29

**Authors:** Bhavya S. Doshi, Bagirath Gangadharan, Christopher B. Doering, Shannon L. Meeks

**Affiliations:** 1 Emory University School of Medicine, Atlanta, Georgia, United States of America; 2 Aflac Cancer Center and Blood Disorders Service, Children’s Healthcare of Atlanta and Emory University, Atlanta, Georgia, United States of America; Vanderbilt University, United States of America

## Abstract

Development of inhibitory antibodies to coagulation factor VIII (fVIII) is the primary obstacle to the treatment of hemophilia A in the developed world. This adverse reaction occurs in 20–30% of persons with severe hemophilia A treated with fVIII-replacement products and is characterized by the development of a humoral and neutralizing immune response to fVIII. Patients with inhibitory anti-fVIII antibodies are treated with bypassing agents including recombinant factor VIIa (rfVIIa). However, some patients display poor hemostatic response to bypass therapy and improved treatment options are needed. Recently, we demonstrated that fVIII inhibitors display widely variable kinetics of inhibition that correlate with their respective target epitopes. Thus, it was hypothesized that for antibodies that display slow rates of inhibition, supplementation of rfVIIa with fVIII would result in improved thrombin generation and be predictive of clinical responses to this novel treatment regimen. In order to test this hypothesis, 10 murine monoclonal antibodies (MAbs) with non-overlapping epitopes spanning fVIII, differential inhibition titers, and inhibition kinetics were studied using a thrombin generation assay. Of the 3 MAbs with high inhibitory titers, only the one with fast and complete (classically defined as “type I”) kinetics displayed significant inhibition of thrombin generation with no improvement upon supplementation of rfVIIa with fVIII. The other two MAbs that displayed incomplete (classically defined as “type II”) inhibition did not suppress the potentiation of thrombin generation by fVIII. All antibodies that did not completely inhibit fVIII activity demonstrated potentiation of thrombin generation by the addition of fVIII as compared to rfVIIa alone. In conclusion, fVIII alone or in combination with rfVIIa corrects the thrombin generation defect produced by the majority of anti-fVIII MAbs better than single agent rfVIIa. Therefore, combined fVIII/rfVIIa therapy may provide better hemostatic control than current therapy in some patients with anti-fVIII inhibitors.

## Introduction

Hemophilia A is an X-linked recessive disorder due to causal mutations in the *F8* gene that lead to absent or decreased factor VIII (fVIII) activity and present phenotypically with abnormal bleeding, both trauma-induced and spontaneous that can be life-threatening. Most patients with hemophilia A are treated by fVIII replacement therapy using either plasma-derived or recombinant products. Approximately 20–30% of patients develop neutralizing IgG-type antibodies against fVIII, which make bleeding more difficult to control clinically. [1], [2], [3], [4] Patients with high-titer inhibitors are treated with bypassing agents such as recombinant activated factor VII (rfVIIa) or activated prothrombin-complex concentrate. However, for reasons that are not well understood, some patients display poor hemostatic response to bypass therapy and improved treatment options are needed. [5], [6].

Anti-fVIII antibody titers classically have been determined by the Bethesda assay. [7] The inhibitor titer, in Bethesda unit (BU) per ml, is defined as the reciprocal of the dilution that produces 50% residual fVIII activity following 2 hour incubation at 37°C. The inhibition of anti-fVIII antibodies is time and temperature dependent, however, the Bethesda assay does not separate antibodies with rapid inhibition from those with slower rates of inhibition. [8] FVIII inhibitors can be either type I or type II inhibitors. Type I inhibitors inhibit fVIII nearly completely while type II inhibitors are incapable of more than 90% inhibition independent of their concentration. [9], [10], [11].

FVIII is a large, plasma glycoprotein and is composed of 6 domains (A1-A2-B-A3-C1-C2) that are characterized based on internal sequence homologies. The majority of inhibitory antibodies are directed at either the A2 or C2 domains of fVIII in either congenital or acquired hemophilia A. [12] Typically, congenital hemophiliacs have a polyclonal response with antibodies recognizing both the A2 and C2 domains, whereas acquired hemophilia patient antibodies typically recognize more limited B cell epitopes consisting of either anti-A2 or anti-C2 antibodies, but not both. [12].

Within the A2 and C2 domain there are non-overlapping B cell epitopes with different functional properties. We have shown that within the C2 domain, inhibitor epitopes can be divided into 2 groups based on functional properties. “Classical” C2 antibodies block binding of fVIII to von Willebrand factor (VWF) and/or phospholipid. “Nonclassical” anti-C2 antibodies are type II inhibitors that generally have 10-fold higher inhibitor titers (BU/mg IgG) than the classical anti-C2 antibodies. In a murine bleeding model, doubling the dose of fVIII corrected the bleeding phenotype in the presence of nonclassical Abs but not classical C2 or a type I anti-A2 MAb. Within the A2 domain, MAbs have inhibitor titers ranging from 0–40,000 BU/mg, and both type I and type II antibodies are represented. The antibodies also vary in the time needed to reach maximum inhibition. [13], [14].

The extent of fVIII inhibition by anti-fVIII antibodies depends on the amount of antibody present and the binding constants for the antibody. Inhibitors also vary in terms of inhibitory titer, time to maximum inhibition, and residual fVIII activity at maximal inhibition. Given our previous work linking the functional characteristics of anti-fVIII antibodies to their fVIII epitope, we analyzed the thrombin generation response in the context of fVIII and/or rfVIIa supplementation in severe hemophilia A plasma spiked with a panel of these biochemically-defined, non-overlapping murine MAbs spanning the A and C domains of fVIII. It was hypothesized that thrombin generation could be potentiated by supplementation with fVIII in the presence of specific inhibitors that display slower inhibition or type II kinetics (i.e. demonstrate incomplete fVIII inactivation during the pharmacokinetic lifetime of rfVIIa).

## Materials and Methods

### Materials

Recombinant full-length fVIII (Kogenate® FS) and rfVIIa (NovoSeven RT®) were gifts from Hemophilia of Georgia (Atlanta, GA). All fVIII deficient plasmas tested (severe hemophilia A plasma) and normal pooled plasma (fVIII: 0.99 U/ml) were obtained from George King Biomedical (Overland Park, KS). Reagents for TGA including thrombin fluorogenic substrate (TGA SUB), tissue factor/phospholipid solution (TGA RC low), and thrombin calibrator (TGA CAL) were purchased from DiaPharma (West Chester, OH).

### Monoclonal Antibodies

MAbs with non-overlapping epitopes across the fVIII protein were made from murine splenic B-cell hybridomas as described previously. [15] One well characterized murine monoclonal antibody, MAb413, was obtained from the Red Cross. BO2C11, a human monoclonal antibody directed against the C2 domain, was obtained from Dr. Marc Jacquemin at the Katholieke Universiteit, Leuven, Belgium. [16], [17] The characteristics of these MAbs are summarized in Table 1.

**Table 1:** Characteristics of anti-fVIII MAbs.

MAb	Domain	Bethesda Titer(BU/mg IgG)	Bethesda Titer(BU/mL)	Kinetics
Panel of MAbs with Non-Overlapping Epitopes				
2–116	A1	0	0	–
1D4	A2	6900	35	Type I
2–54	A2	33000	165	Type II
2–93	A2	4	<1	–
4A4	A2	40000	200	Type I
2–113	A3	Indeterminate	–	Type II
G38	A3	4100	41	Type I
2A9	C1	97	<1	–
I109	C2	1500	15	Type I
2–77	C2	25000	250	Type II
Additional MAbs Tested				
MAb413	A2	21000	210	Type I
BO2C11	C2	20000	200	Type I

### FVIII Inhibitor Assays

Inhibitor titers and residual fVIII activities were measured using Bethesda assay [7] with the modifications previously described. [18] Pooled normal human plasma was used as the source of fVIII activity. The antibodies were classified as type II if the residual fVIII activity was greater than 10% at saturating levels of antibody. [13], [19].

### Epitope Mapping

Domain-specific anti-fVIII MAb epitope mapping was carried out by direct ELISA using recombinant hybrid human/porcine constructs as previously described. [20] Briefly, MAbs were serially diluted in blocking buffer and placed on half-area 96 well plates using purified single human domain hybrid fVIII proteins as test antigens and recombinant human fVIII and BDD porcine fVIII as positive control antigens. ELISA titers were defined as the reciprocal plasma dilution for each test antigen that resulted in an A_405_ value of 0.3. Values for single human domain hybrids were compared with the human and porcine controls to determine domain specificity.

### Rate of Inhibition of fVIII

The rate of inhibition by the MAbs was determined using a one-stage coagulation assay. FVIII deficient plasma was spiked with recombinant fVIII and mixed 1∶1 with MAbs to achieve final volume of 40 µl and final concentrations of 1 U/ml fVIII (∼1 nM) and 5 µg/ml MAb (∼30 nM). These mixtures were incubated for 0, 5, 15, 30, 60, 90 and 120 minutes and one-stage coagulation assays were performed at each time point to determine residual fVIII activity.

### Thrombin Generation Assays

FVIII-dependent thrombin generation in plasma at 37°C following initiation of coagulation with tissue factor and phospholipid was measured using the fluorometric thrombin substrate Z-Gly-Gly-Arg-AMC. FVIII deficient plasma (George King) was spiked with recombinant fVIII, rfVIIa or both and mixed 1∶1 with MAb in fVIII deficient plasma to final concentrations of 1 U/ml fVIII, 2.25 µg/ml fVIIa and 5, 10 or 50 µg/ml MAb. Samples were placed on 96-well MicroFluor 2B plates (ThermoScientific) and TGA RC low (low concentration of phospholipid micelles and pM recombinant tissue factor) and TGA substrate were added just before beginning the assay. Fluorescence was read for 60 minutes using a BioTek SynergyMx (model SMABTL) fluorescence reader. Data were analyzed using evaluation software provided by the manufacturer. The parameters analyzed include ETP, peak thrombin concentration, time to peak thrombin, lag time and index velocity. The ETP is defined as the area under the thrombin generation curve. Lag time is defined as the time to one-sixth of the peak thrombin concentration of a sample. Index velocity is defined by the peak thrombin concentration divided by the difference between time to peak and lag time. The TGA was carried out twice for each MAb: (1) fVIII and MAb were combined and the assay run immediately; (2) fVIII and MAb were incubated together for 1 hour prior to running the assay.

## Results

### Selection and Classification Anti-fVIII MAbs

**Figure 1:**
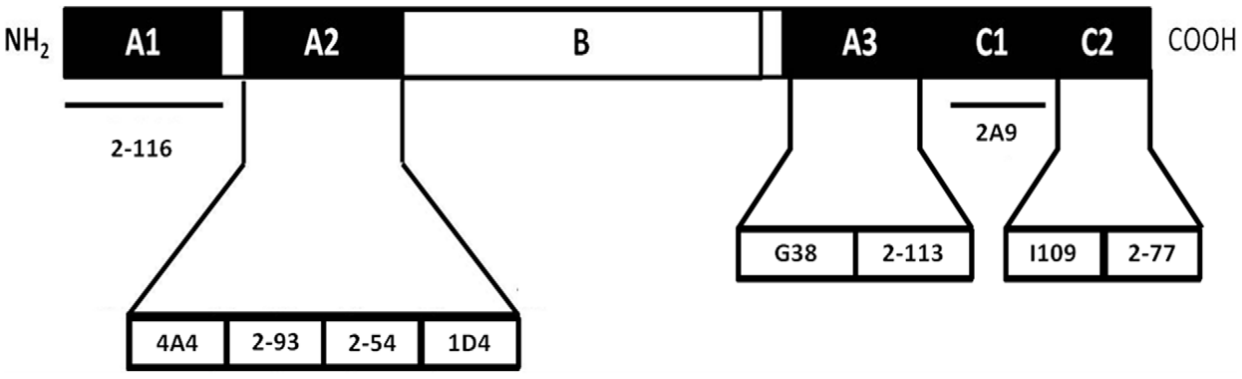
Epitope map of non-overlapping MAbs. The relative epitopes of anti-fVIII MAbs are shown. MAbs 4A4, 2–54, 1D4 and 2–93 target non-overlapping epitopes in the A2 domain. MAbs G38 and 2–113 target non-overlapping epitopes in the A3 domain at residues 1690–1817 and 1818–1916, respectively. MAbs I109 and 2–77 target non-overlapping epitopes in the C2 domain. [13].

A panel of 10 murine MAbs was selected to target non-overlapping B-cell epitopes distributed throughout the A and C domains of human fVIII. MAbs targeting the B domain were not selected for the current study since the B domain has no known role in cofactor function (Figure 1, Table 1). Each MAb was classified based on domain specificity, inhibitor titer, inhibitor kinetics, and time to maximum inhibition of fVIII activity. Inhibitor titers ranged from non-inhibitory to 40,000 BU/mg. Based on the inhibitor titer data obtained at a MAb concentration of 5 µg/ml, the MAbs could be divided into 3 groups. Group 1 contained 4 MAbs that were low titer inhibitors <5 BU/ml or non-inhibitory. Groups 2 and 3 contained the remaining 6 MAbs which were all high titer inhibitors (>5 BU/ml). Group 2 had MAbs with titers between 15 and 41 BU/ml, and group 3 had MAbs with titers greater than 164 BU/ml. The group 1 inhibitors included 2–116 (A1 domain epitope), 2–93 (A2 domain epitope), 2–113 (A3 domain epitope), and 2A9 (C1 domain epitope). The exact titer of 2–113 could not be determined because it is a type II inhibitor with maximum inhibition in the Bethesda assay of 35% that was reached following 15 minute incubation. The remaining low titer MAbs inhibited to a lesser extent resulting in greater than 70% residual fVIII activity after 1 hour incubation.

The group 2 MAbs included 1D4 (A2 domain epitope), G38 (A3 domain epitope), and I109 (C2 domain epitope). All 3 are type I inhibitors. Both 1D4 and G38 rapidly inhibited fVIII activity reaching maximum inhibition at 5 and 15 minutes, respectively. I109 displayed a slower rate of inhibition reaching maximum inhibition at 60 minutes. I109 is a classical C2 domain targeting antibody that is known to compete with VWF for binding to fVIII.

There were 3 group 3 MAbs including 2–54 (A2 domain epitope), 4A4 (A2 domain epitope), and 2–77 (C2 domain epitope). MAb 4A4 is a type I inhibitor that reaches maximum inhibition within 5 minutes. MAb 2–54 and 2–77 are type II inhibitors that rapidly, but incompletely inhibit fVIII activity in vitro.

### Thrombin Generation Assays in fVIII Deficient Plasma Supplemented with rfVIIa ± fVIII

**Figure 2:**
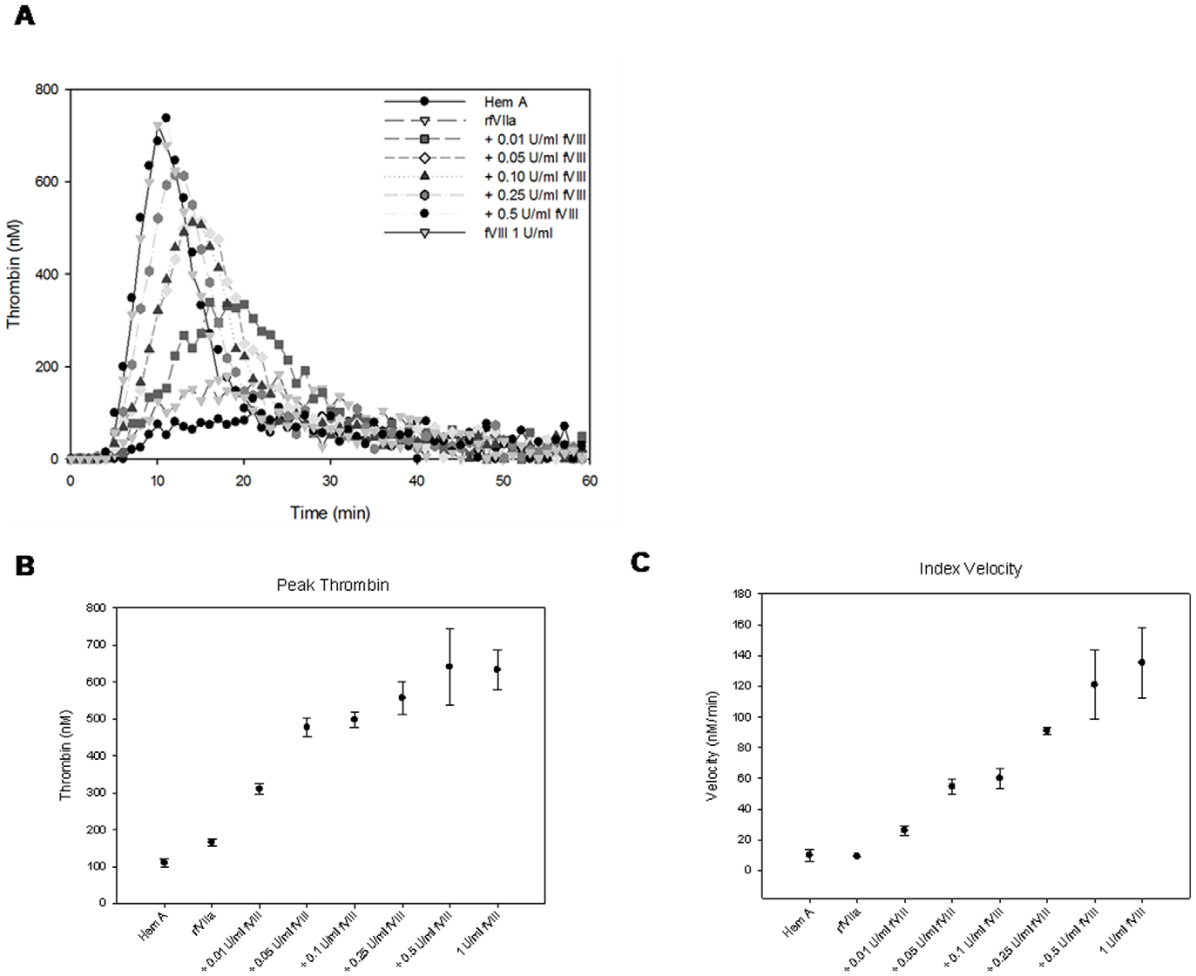
Amount of fVIII needed to restore thrombin generation of rfVIIa. Varying concentrations of fVIII were mixed 1∶1 with rfVIIa at a final concentration of 2.25 µg/ml to determine the level of fVIII activity necessary for restoration of thrombin generation of rfVIIa to fVIII at 1 U/ml. The resulting data was transformed using manufacturer’s software to yield thrombin generation curves (A), peak thrombin concentration (B) and index velocity (C). Error bars represent sample standard deviation.

Compared to fVIII supplementation, rfVIIa supplementation alone produces a lower peak thrombin concentration and a smaller ETP when added to fVIII-deficient plasma in the TGA (Figure 2A). As proof of concept that fVIII supplementation would potentiate thrombin generation by rfVIIa, we determined the amount of fVIII necessary to achieve equivalence between the thrombin generation of rfVIIa with fVIII and fVIII alone at 1 U/ml. As little as 0.01 U/ml fVIII improved thrombin generation of rfVIIa in fVIII-deficient plasma in the TGA. The ETP of rfVIIa supplemented with fVIII approached equivalence to 1 U/ml fVIII (6520±450 nM·min) at 0.01 U/ml fVIII (5680±190 nM.min) and was higher with 0.05 U/ml fVIII (6720±710 nM·min). The peak thrombin achieved was equivalent to 1 U/ml fVIII alone (630±50 nM) at 0.1 U/ml (500±20 nM) fVIII and rfVIIa and was higher at 0.5 U/ml fVIII with rfVIIa (640±110 nM) (Figure 2B). The index velocity achieved equivalence at 0.5 U/ml fVIII supplementation to rfVIIa as well (Figure 2C).

### Thrombin Generation in the Presence of Anti-fVIII MAbs

TGA curves and their respective parameter values were determined for each MAb (5 µg/ml) in samples containing 1 U/ml fVIII, 2.25 µg/ml rfVIIa or both. Thrombin generation was measured either immediately after adding the inhibitor or after 1 hr incubation. Severe hemophilia A plasma with no inhibitor was used as the negative control and thrombin generation parameters (ETP, peak thrombin and index velocity) were normalized to this control. rfVIIa generated similar parameter values for all samples with or without MAbs at the immediate (ETP 59%±16%, peak thrombin 16%±5%, and index velocity 6%±2% fVIII) and 1 hr (ETP 67%±11%, peak thrombin 23%±3%, and index velocity 14%±7% fVIII) time points.

**Figure 3:**
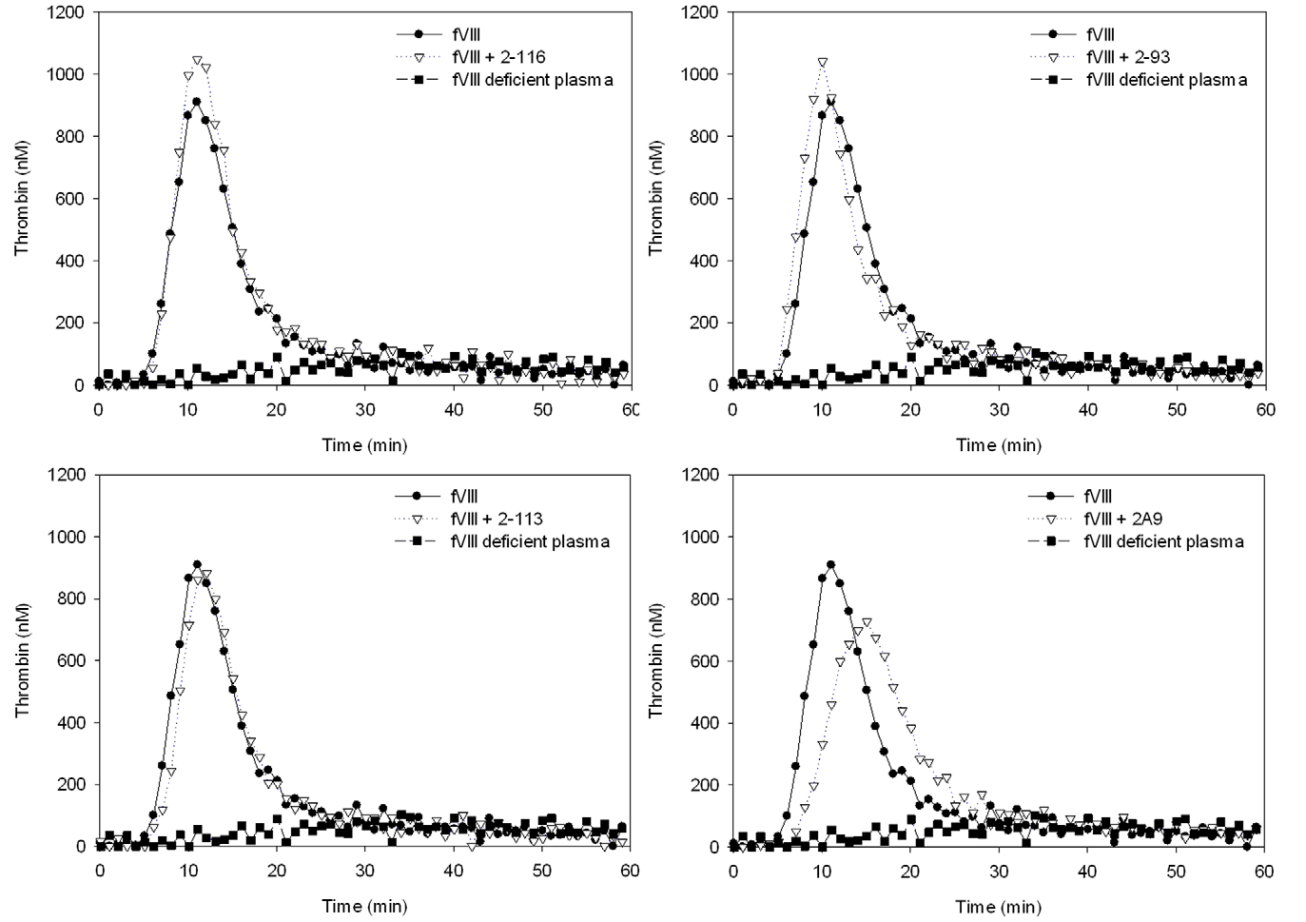
Inhibition of thrombin generation by group 1 anti-fVIII MAbs. Thrombin generation curves are shown for fVIII deficient plasma (negative control), fVIII deficient plasma with 1 U/ml of fVIII (positive control) and 1 U/ml of fVIII in the presence of 5 µg/ml of specified group 1 MAb. Data represent mean ± sample standard deviation for 3 experiments where duplicates of each measurement were taken and averaged.

Comparing fVIII alone to fVIII with MAb, 3 of 4 group1 MAbs (2–116, 2–113, and 2–93) produced no change in the ETP at the immediate and 1 hr time points (Figure 3). MAb 2A9 affected no change in ETP but did decrease peak thrombin at 1 hour to 70% of the no MAb control. Supplementation of rfVIIa with fVIII produced no improvement in ETP over fVIII alone (Table 2). The peak thrombin concentration and index velocity also were higher in the presence of fVIII than rfVIIa alone. By all parameters tested, the group 1 minimally inhibitory MAbs allowed more thrombin generation in the presence of fVIII than rfVIIa.

**Table 2:** TGA parameters for anti-fVIII MAbs.

MAb	Domain	Thrombin Generation Parameter	No Incubation			1 Hour Incubation		
			fVIII	rfVIIa	fVIII + fVIIa	fVIII	rfVIIa	fVIII + fVIIa
None	–	ETP (ratio)	1.0±0.0	0.6±0.2	0.7±0.2	1.0±0.0	0.7±0.1	1.3±0.3
		Peak (ratio)	1.0±0.0	0.2±0.1	0.7±0.2	1.0±0.0	0.2±0.0	1.4±0.3
		Lag Time (min)	7.6±0.9	7.6±1.8	6.9±1.0	7.8±1.9	6.8±1.5	6.3±1.0
		Peak Time (min)	11.8±1.3	24.6±3.5	12.3±2.9	14.3±1.0	24.8±3.5	11.4±0.8
		Velocity (ratio)	1.0±0.0	0.1±0.0	0.7±0.3	1.0±0.0	0.1±0.1	1.7±0.4
2–116	A1	ETP (ratio)	1.2±0.2	0.6±0.1	1.0±0.2	1.1±0.0	0.7±0.0	1.1±0.2
		Peak (ratio)	1.2±0.1	0.2±0.1	0.9±0.1	1.1±0.1	0.3±0.0	1.4±0.2
		Lag Time (min)	7.9±0.8	8.1±0.0	5.8±0.3	6.9±1.6	6.3±1.8	5.8±1.3
		Peak Time (min)	11.6±1.0	26.9±6.7	9.4±0.6	13.3±1.0	23.6±5.4	10.3±1.6
		Velocity (ratio)	1.3±0.3	0.1±0.0	1.0±0.2	1.1±0.1	0.1±0.1	1.9±0.5
1D4	A2	ETP (ratio)	0.9±0.2	0.6±0.2	0.8±0.2	0.6±0.1	0.6±0.0	0.8±0.2
		Peak (ratio)	0.6±0.2	0.2±0.1	0.6±0.1	0.3±0.0	0.2±0.0	0.4±0.1
		Lag Time (min)	9.4±0.6	8.3±0.8	7.3±0.6	9.6±3.1	7.1±1.3	6.6±1.7
		Peak Time (min)	18.1±2.3	27.1±5.2	13.9±1.0	26.9±5.0	25.6±6.1	21.8±3.3
		Velocity (ratio)	0.4±0.2	0.1±0.0	0.4±0.1	0.1±0.1	0.1±0.1	0.2±0.1
2–54	A2	ETP (ratio)	1.0±0.1	0.6±0.2	1.1±0.4	1.0±0.1	0.7±0.1	1.2±0.1
		Peak (ratio)	1.0±0.1	0.2±0.1	0.9±0.1	1.2±0.2	0.3±0.1	1.5±0.3
		Lag Time (min)	10.3±1.3	7.1±1.5	6.8±1.0	10.3±2.8	6.9±1.8	5.9±1.3
		Peak Time (min)	16.6±1.3	25.6±4.4	13.1±0.9	18.1±3.1	22.6±4.4	13.4±2.3
		Velocity (ratio)	0.6±0.2	0.1±0.0	0.6±0.1	0.9±0.2	0.2±0.1	1.3±0.5
2–93	A2	ETP (ratio)	1.1±0.1	0.6±0.1	1.1±0.2	1.1±0.1	0.6±0.1	1.3±0.2
		Peak (ratio)	1.2±0.1	0.1±0.0	1.1±0.1	1.5±0.2	0.2±0.0	1.8±0.4
		Lag Time (min)	7.1±1.0	6.8±1.9	5.8±0.3	7.3±1.5	7.1±0.9	5.6±1.3
		Peak Time (min)	10.1±0.9	26.1±3.1	8.9±0.8	11.3±1.3	23.3±5.9	8.9±1.3
		Velocity (ratio)	1.6±0.3	0.1±0.0	1.4±0.3	2.3±0.2	0.2±0.1	3.3±1.0
4A4	A2	ETP (ratio)	0.0±0.0	0.6±0.0	0.8±0.1	0.1±0.1	0.6±0.2	0.7±0.2
		Peak (ratio)	0.0±0.0	0.2±0.0	0.2±0.1	0.0±0.0	0.2±0.1	0.3±0.0
		Lag Time (min)	13.1±2.8	8.3±1.2	7.6±1.0	10.8±3.2	7.1±1.3	6.4±0.8
		Peak Time (min)	44.8±11.0	26.3±3.0	25.1±4.3	38.9±15.4	25.4±5.3	23.4±5.9
		Velocity (ratio)	0.0±0.0	0.1±0.0	0.1±0.0	0.1±0.1	0.1±0.1	0.2±0.1
2–113	A3	ETP (ratio)	0.9±0.1	0.5±0.1	1.3±0.5	1.0±0.1	0.8±0.3	0.7±0.1
		Peak (ratio)	0.9±0.1	0.1±0.0	1.2±0.5	1.1±0.1	0.3±0.2	1.0±0.1
		Lag Time (min)	9.4±2.8	5.8±3.6	5.9±0.8	7.9±2.1	7.6±1.7	5.3±0.8
		Peak Time (min)	10.9±2.0	25.9±1.6	9.8±0.6	14.6±2.2	23.1±6.5	10.1±0.5
		Velocity (ratio)	1.0±0.3	0.0±0.0	1.4±0.7	1.0±0.3	0.3±0.3	1.4±0.1
G38	A3	ETP (ratio)	0.9±0.2	0.6±0.2	1.4±0.4	0.8±0.1	0.7±0.2	0.6±0.3
		Peak (ratio)	0.7±0.1	0.1±0.1	1.0±0.3	0.5±0.0	0.3±0.1	0.6±0.3
		Lag Time (min)	9.1±0.5	8.1±1.0	6.4±0.8	10.3±2.8	6.9±2.1	5.9±1.9
		Peak Time (min)	15.4±0.6	28.4±6.7	11.9±1.0	24.4±2.5	27.6±6.9	17.1±1.8
		Velocity (ratio)	0.6±0.3	0.1±0.0	0.9±0.4	0.3±0.0	0.2±0.1	0.4±0.3
2A9	C1	ETP (ratio)	1.1±0.2	0.7±0.1	1.3±0.3	0.9±0.0	0.8±0.3	0.7±0.3
		Peak (ratio)	0.8±0.2	0.2±0.0	1.0±0.4	0.7±0.1	0.3±0.2	0.8±0.3
		Lag Time (min)	8.8±0.8	7.9±1.0	6.1±0.0	8.6±2.6	6.8±1.9	5.3±1.2
		Peak Time (min)	15.8±0.6	23.6±1.8	12.3±0.3	19.4±3.1	22.9±6.4	13.8±1.3
		Velocity (ratio)	0.5±0.2	0.1±0.0	0.7±0.3	0.5±0.1	0.2±0.2	0.7±0.3
I109	C2	ETP (ratio)	0.9±0.5	0.7±0.2	1.0±0.2	0.8±0.1	0.8±0.1	0.8±0.2
		Peak (ratio)	0.6±0.3	0.2±0.0	0.8±0.2	0.6±0.1	0.3±0.1	0.9±0.3
		Lag Time (min)	9.3±2.0	8.4±1.2	6.6±1.3	9.3±2.5	6.9±2.3	6.9±1.5
		Peak Time (min)	18.3±1.0	25.8±6.0	13.9±0.8	21.1±2.6	28.3±3.8	15.3±2.3
		Velocity (ratio)	0.3±0.2	0.1±0.0	0.5±0.2	0.4±0.1	0.1±0.1	0.7±0.3
2–77	C2	ETP (ratio)	0.9±0.3	0.6±0.2	1.0±0.3	0.9±0.1	0.7±0.1	0.8±0.2
		Peak (ratio)	0.6±0.2	0.2±0.0	0.8±0.3	0.7±0.1	0.3±0.1	0.9±0.3
		Lag Time (min)	10.4±0.8	8.6±1.0	7.1±0.5	9.8±2.0	7.6±1.8	6.3±0.6
		Peak Time (min)	17.8±0.8	27.9±7.5	13.6±0.5	19.1±1.8	24.4±7.6	14.8±1.8
		Velocity (ratio)	0.4±0.2	0.1±0.0	0.6±0.2	0.6±0.1	0.2±0.1	0.8±0.4

ETP, peak thrombin and index velocity are presented as ratios compared to fVIII deficient plasma supplemented with 1 U/ml fVIII in the absence of any anti-fVIII MAb.

ETP – endogenous thrombin potential.

**Figure 4:**
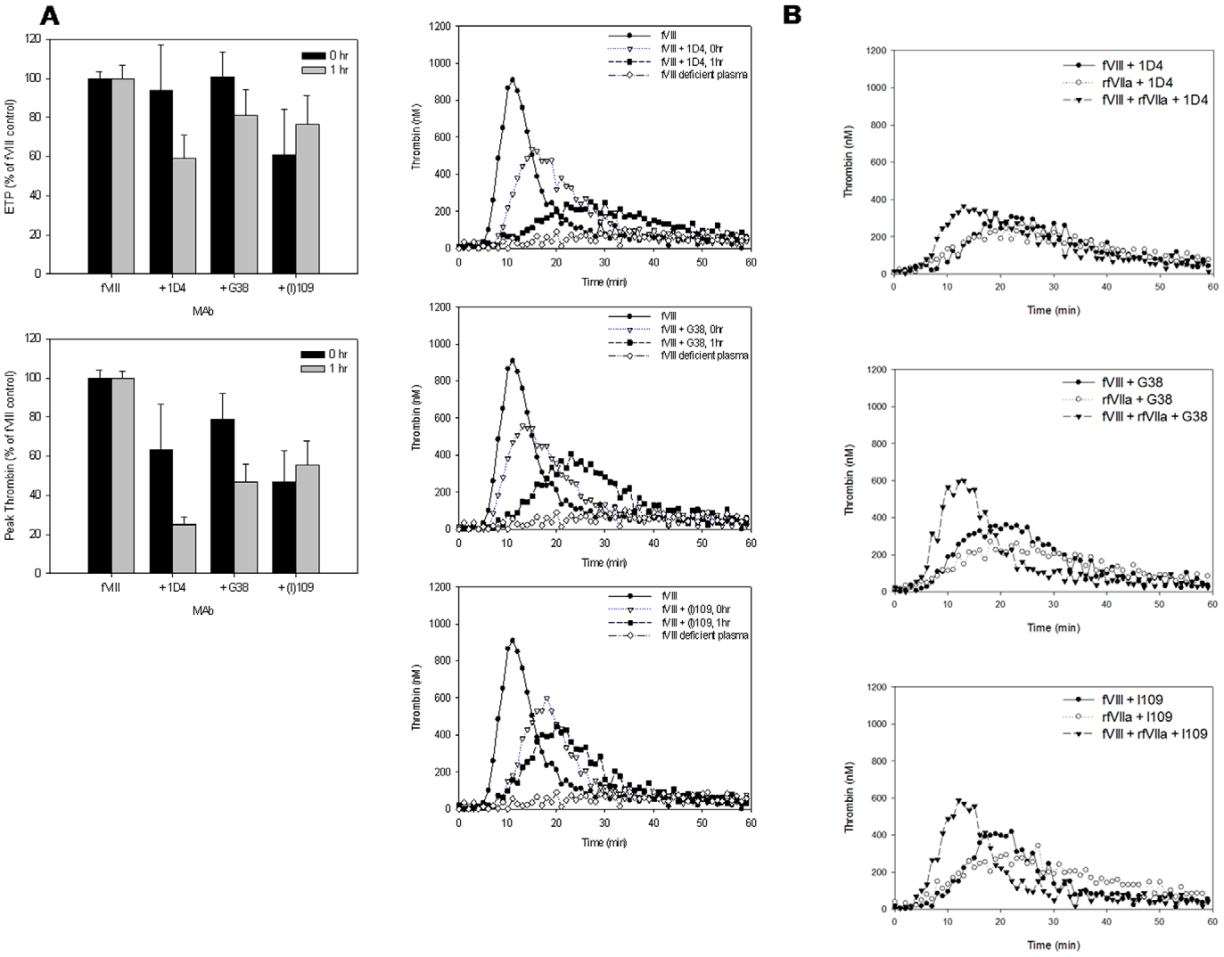
Time dependent inhibition of thrombin generation by group 2 anti-fVIII MAbs. Thrombin generation curves are shown for fVIII deficient plasma (negative control), fVIII deficient plasma with 1 U/ml of fVIII (positive control) and 1 U/ml of fVIII in the presence of 5 µg/ml of specified group 2 MAb at both the immediate and 1 hr time points (A) Thrombin generation curves comparing fVIII alone, rfVIIa alone, and fVIII and rfVIIa in the presence of MAb at the 1 hr time points (B). Graphs shown are representative of 3 independent experiments.

The group 2 MAbs (1D4, G38 and I109) with inhibitor titers between 15 and 41 BU/ml all are type I inhibitors that demonstrate variable time to maximum inhibition of 5, 15, and 60 minutes, respectively. Each of these MAbs displayed increased inhibition of thrombin generation over time in the TGA (Table 2, Figure 4A). After 1 hr incubation in the presence of the A2 domain epitope-targeting MAb, 1D4, the residual fVIII activity was 3% and the ETP was similar whether or not fVIII or rfVIIa alone were present but was higher when both were present (83% of fVIII control). The peak thrombin and index velocity in the presence of fVIII alone were inhibited to the levels observed in the presence of rfVIIa alone and were not elevated to fVIII control levels by co-addition of fVIII and rfVIIa. G38, an A3 domain epitope targeting MAb, displayed a higher ETP, peak thrombin and index velocity with fVIII alone as compared to rfVIIa alone even after 1 hr incubation. I109, a C2 domain epitope targeting MAb, showed the same ETP with fVIII, rfVIIa alone, or fVIII + rfVIIa at the 1 hr time point (∼80% of control). FVIII alone produced a higher peak thrombin and index velocity than rfVIIa alone, but the combination was superior to either factor alone. Overall, these group 2 MAbs are time dependent inhibitors and show improvement in some but not all parameters when fVIII and rfVIIa are combined.

**Figure 5:**
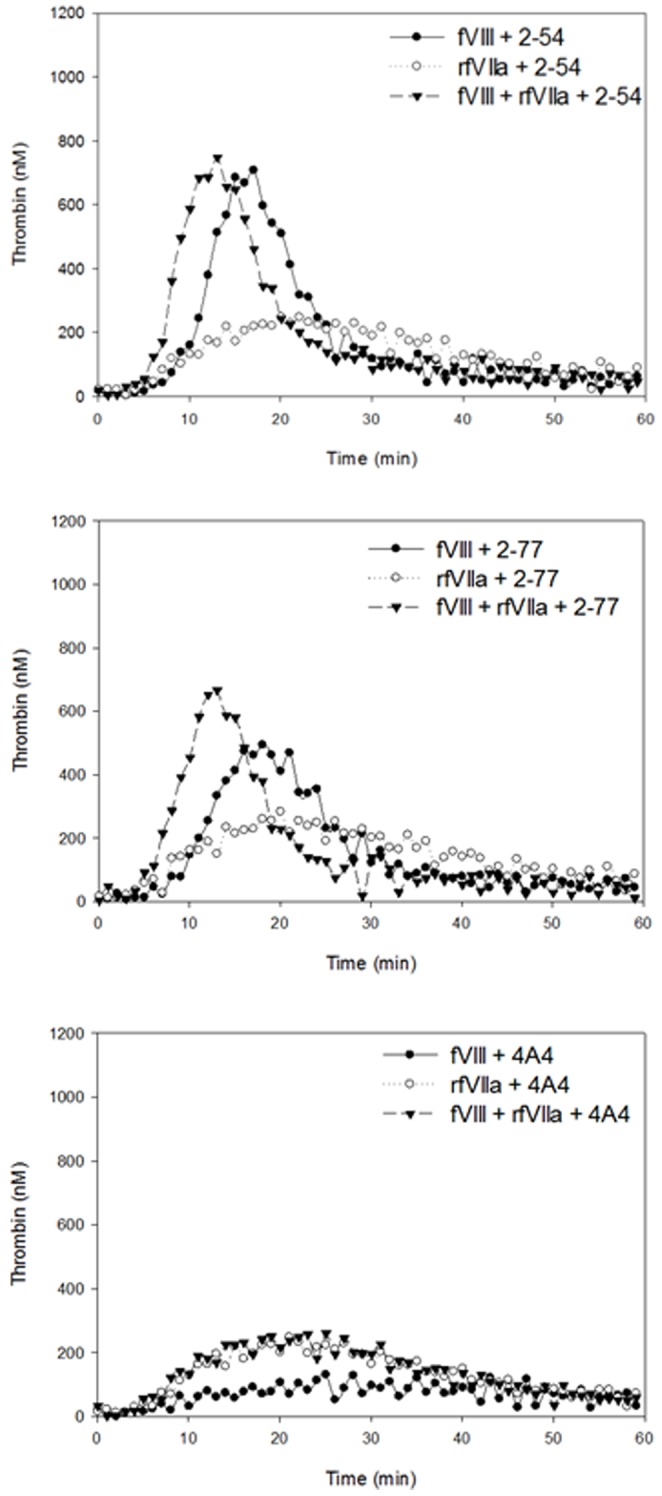
Thrombin generation in the presence of group 2 anti-fVIII MAbs. Thrombin generation curves are shown comparing fVIII alone, rfVIIa alone, and fVIII and rfVIIa in the presence of MAb at the 1 hr time points. Graphs shown are representative of 3 independent experiments.

Of the group 3 MAbs (inhibitory titers greater than 164 BU/ml), the 2 type II MAbs, 2–54 and 2–77, responded differently than the type I MAb 4A4. Addition of the high-titer type II inhibitory MAbs, 2–54 and 2–77, to fVIII had little effect on thrombin generation even after 1 hr incubation, while the high titer type I inhibitor, 4A4, inhibited thrombin generation almost completely (Table 2, Figure 5). Compared to no MAb control, the ETPs of these MAbs at the immediate and 1 hr incubation time points were 101% and 103% for 2–54, 90% and 90% for 2–77 and 5% and 11% for 4A4, respectively. With the addition of rfVIIa to fVIII, the ETPs for 2–54 and 2–77 did not change at the immediate or 1 hr incubation time points. MAb 2–77 inhibited the peak thrombin in the presence of fVIII alone to 64% and 74% of the fVIII only control. Addition of rfVIIa improved peak thrombin to 84% and 88% after 0 and 1 hr incubations, respectively. The lag time was longer with fVIII in the presence of either 2–54 or 2–77. However, this delay was corrected with addition of rfVIIa to fVIII. Supplementation of rfVIIa with fVIII increased the ETP of 4A4 over rfVIIa alone at the immediate (81% of fVIII), but not the 1 hr incubation time point. Despite similar inhibitor titers in these MAbs, the type I and II MAbs demonstrated dramatically different effects on thrombin generation.

There is known wide variability in the baseline thrombin generation in both fVIII deficient plasma and normal patient plasma. Given this variability and the relatively high thrombin generation in the initial fVIII deficient patient plasma used two additional commercial fVIII deficient plasmas were tested. For each fVIII deficient plasma all MAbs with non-overlapping epitopes were tested under the same conditions as described previously. Similar relative results were seen despite thrombin generation (ETP) of fVIII deficient plasma alone ranging from 626 nM·min to 2432 nM·min (data not shown).

### Analysis of Additional High-titer Type I MAbs

The high-titer type I inhibitor 4A4 was the only MAb that inhibited ETP, peak thrombin and index velocity of thrombin generation to a level that could not be increased by fVIII supplementation in the presence of rfVIIa. Therefore, we tested 2 additional high-titer type I MAbs. MAb413 is a well-characterized murine A2 domain targeting MAb with a titer of 21,000 BU/mg [21], [22] that targets a B-cell epitope, which overlaps the epitope targeted by 4A4. Similar to 4A4, it completely inhibited thrombin generation by 1 hr in the presence of fVIII alone and there was no improvement upon supplementation of rfVIIa with fVIII. BO2C11 is a type I anti-C2 human MAb that blocks fVIII binding to VWF and phospholipid, overlaps murine MAb I109, but has an inhibitor titer that is almost 20-fold higher. [13] BO2C11 has a slower rate of inhibition than 4A4 and MAb413 and inhibits thrombin generation to a lower extent than MAb413 or 4A4 (ETP 48%±14% fVIII, peak thrombin 19%±5% fVIII and index velocity 7%±4% fVIII at the immediate time point). In the presence of BO2C11, supplementation of rfVIIa with fVIII improved thrombin generation more so at the immediate (ETP 81%±20%, peak thrombin 44%±6%, index velocity 22%±7%) than at the 1 hr time point (ETP 64%±32%, peak thrombin 34%±16%, index velocity 21%±12%).

### Effect of MAb Concentration on Thrombin Generation

Given that the group 2 type I MAbs, I109 and G38, displayed incomplete inhibition at a concentration of 5 µg/ml over 1 hr, the MAb concentrations were increased to 10 and 50 µg/ml and the TGA experiments were repeated. I109 continued to allow higher ETP, peak thrombin and index velocity after supplementation with rfVIIa and fVIII at both the immediate and 1 hr incubation times. G38 also showed improved thrombin generation with rfVIIa and fVIII both immediately and following incubation at the 10 µg/ml dose but at the 50 µg/ml dose it showed improvement immediately (ETP 20% with fVIII alone, 70% with rfVIIa + fVIII) but not following 1 hr incubation. At the highest concentration, group 1 MAb 2A9, which showed minimal inhibition at 5 µg/ml, did not permit an increase in thrombin generation upon fVIII supplementation of rfVIIa (Tables S1 and S2).

Lastly, the concentrations of the type II high titer MAbs 2–54 and 2–77 were increased to 10 or 50 µg/ml. At both concentrations, the thrombin generation in the presence of 2–54 and fVIII was not different than the fVIII control in terms of ETP, peak thrombin or index velocity at 0 hr or 1 hr incubation. MAb 2–77 showed similar trends at 10 and 50 µg/ml as at 5 µg/ml with decreases in peak thrombin and index velocity with fVIII alone and improvement when fVIII was combined with fVIIa. The ETP decreased slightly for the 50 µg/ml sample only at the immediate time point. These results indicate that increasing the MAb concentration of type II MAbs has little to no effect on thrombin generation (Tables S1 and S2).

**Figure 6:**
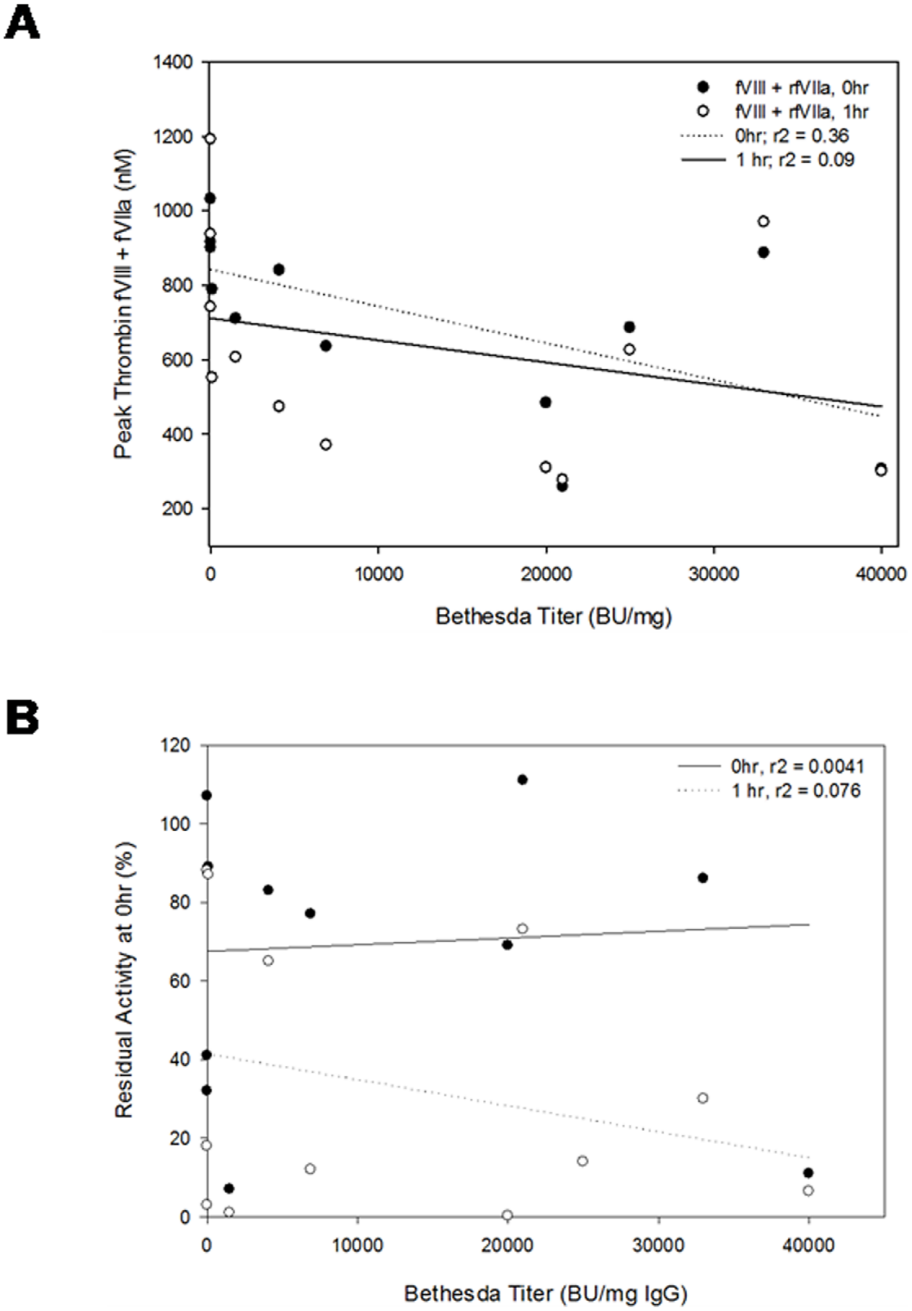
Influence of inhibitory titer on residual fVIII activity and peak thrombin generation. The inhibitor titer for each MAb was compared with the peak thrombin generation of fVIII alone or fVIII + rfVIIa at the immediate (solid circles, r^2^ = 0.36) or 1 hr (open circles, r^2^ = 0.09) time points (A.) and with residual fVIII activity at the immediate (solid circles, r^2^ = 0.004) or 1 hr (r^2^ = 0.07) time points (B.).

For the entire panel of antibodies at 5 µg/ml dose there was no correlation between inhibitor titer and peak thrombin either immediately or after 1 hr incubation in the presence of fVIII alone or with the combination of fVIII and fVIIa. Furthermore, there also was no correlation between inhibitor titer and residual fVIII activity (Figure 6). These results suggest that inhibitor titer alone cannot be used to predict the amount of thrombin generated in response to fVIII alone or combinations of fVIII and rfVIIa.

## Discussion

The ability to predict response to fVIII and bypassing agents is needed for individual patients with hemophilia A complicated by fVIII inhibitors. Physicians treating high-titer inhibitor patients typically base treatment decisions on the fVIII inhibitor titer as measured by the Bethesda assay. Patients with inhibitor titers greater than 10 BU/ml are regarded as unlikely to respond to fVIII administration and are treated with bypassing agents such as rfVIIa and aPCCs. However, a subset of patients may not have adequate hemostasis with bypassing agents and other treatment options are needed. A recent report highlighted the potential that combinations of fVIII and bypassing agents may result in higher levels of thrombin generation than either agent individually but did not address the mechanisms behind which patients responded. [23] It should be noted that some inhibitor patients, particularly those not on immune tolerance with fVIII, may have a memory B-cell response to fVIII infusions potentially boosting inhibitor titers. Recent data in our laboratory has shown that the epitope specificity and inhibitor kinetics within the C2 domain are more important than inhibitor titer in predicting the response to fVIII. [14] Additionally in the absence of anti-fVIII inhibitors, even 1% fVIII supplementation to rfVIIa improved the ETP, peak thrombin and index velocity of thrombin generation in hemophilia A plasma. Given these observations and our knowledge of characteristics of non-overlapping epitopes on the other immunodominant domain, the A2 domain, we hypothesized that antibodies to different epitopes would respond differently to combinations of fVIII and rfVIIa and that this response would be related to the titer, the type of inhibitor, and the rate of inhibition.

The panel of MAbs studied targeted all functional domains of fVIII and varied in epitope, inhibitor titer, inhibitor type, and rate of inhibition. Using this diverse panel, it was possible to investigate thrombin generation in the presence of MAb and fVIII and/or rfVIIa. Given the initial result demonstrating that even 1% (of normal human level) residual fVIII activity increased thrombin generation in the presence of rfVIIa (Figure 2), it was predictable that, in the presence of the majority of MAbs, increased thrombin generation occurred when fVIII was supplemented to rfVIIa. While prior studies have shown that fVIII with bypassing agents can potentiate thrombin generation in patients with inhibitors, the doses of fVIII were supra-therapeutic. [23] In the current study, the dose of fVIII used was the *in vitro* equivalent of 50 U/kg, which is a typical treatment dose used in hemophilia A patients without inhibitors.

Only the overlapping, high-titer, type I MAbs targeting the A2 domain, 4A4 and MAb413, displayed nearly complete inhibition at the immediate time point indicating that the epitopes blocked by these antibodies are essential to thrombin generation. In contrast, the MAbs targeting the C2 domain incompletely inhibited thrombin generation and their inhibition could be overcome with fVIII + rfVIIa supplementation. This set includes B02C11, which like 4A4 and MAb413 is a type I inhibitor and has similar specific inhibitory activity. B02C11 inhibits the binding of fVIII to phospholipid and VWF [13] and this competition with VWF in plasma may contribute to the slower time to maximum inhibition seen with B02C11. In addition, the 2 type II MAbs with similar high titers, 2–54 (A2 domain targeting) and 2–77 (C2 domain targeting) showed minimal inhibition to thrombin generation with fVIII alone, which was overcome by rfVIIa and fVIII supplementation. The differential responses between high-titer type I MAbs to different epitopes, 4A4 and MAb413 versus BO2C11, as well as the differential responses to high titer type I and high titer type II MAbs underscore the lack of correlation between inhibitor titer and response to fVIII supplementation. This result suggests that the mechanism of fVIII inhibition of different epitopes may play a role in the response of various therapeutic interventions in the treatment of inhibitor patients and that the ability to identify patients with antibodies to the A2 epitope shared by 4A4 and MAb413 may allow physicians to identify patients not likely to respond to fVIII supplementation. For patients on immune tolerance therapy the knowledge of their epitope spectrum and how it is changing over time may allow physicians to identify patients in whom the fVIII infusions given for immune tolerance may also result in improved hemostasis.

Recent data have shown that TGA may be useful in predicting response to bypassing therapy. [23], [24], [25], [26], [27], [28] However, there is some debate on which parameters are the most important. ETP has been used to determine bleeding severity in hemophilia A patients. [25] Others also have shown that in patients with hemophilia A without inhibitors, peak thrombin concentration was a more sensitive indicator of clinical bleeding risk. [24] In the current study, 2–77, a high-titer type II nonclassical MAb targeting the C2 domain, which also displays pathogenicity in a murine *in vivo* bleeding model, did not alter ETP but did have some inhibition of peak thrombin and index velocity and served to increase the lag time. This suggests that further experimentation is needed to determine which indices, or combination of indices, are predictive of hemostatic response in inhibitor patients.

In summary, we find that thrombin generation in the presence of fVIII does not correlate with the inhibitor titer of anti-fVIII MAbs. This lack of correlation points to the need to find better laboratory tests that can predict response both in vitro and in vivo. Characteristics of the inhibitor such as time to maximum inhibition and type II inhibition lead to residual fVIII activity that can either alone or in combination with rfVIIa improve thrombin generation. Within the group of antibodies targeting the C2 domain of fVIII, we found that antibody characteristics and bleeding phenotypes in a murine *in vivo* bleeding model were similar among MAbs with overlapping epitopes. [13], [14] In addition, we provide evidence that the A2 epitope blocked by the overlapping MAbs 4A4 and MAb413 is essential to the thrombin generation potentiation provided by fVIII. This further suggests that clinical determination of epitope specificity has the potential to predict response to fVIII supplementation better than inhibitor titer alone. Given that patients typically have polyclonal responses to fVIII, epitope mapping of patient plasmas to these sites could help determine the response to fVIII supplementation on top of rfVIIa therapy and may provide a novel approach towards the treatment of hemophilia A complicated by anti-fVIII inhibitors.

## Supporting Information

Table S1 **Thrombin generation parameters for MAbs at 10 µg/ml.** ETP, peak thrombin and index velocity are presented as ratios compared to fVIII deficient plasma supplemented with 1 U/ml fVIII in the absence of any anti-fVIII MAb. ETP – endogenous thrombin potential.
(DOC)

Table S2 **Thrombin generation parameters for MAbs at 50 µg/ml.** ETP, peak thrombin and index velocity are presented as ratios compared to fVIII deficient plasma supplemented with 1 U/ml fVIII in the absence of any anti-fVIII MAb. ETP – endogenous thrombin potential.
(DOC)
