# Supplementary material for: Potentiation of Thrombin Generation in Hemophilia A Plasma by Coagulation Factor VIII and Characterization of Antibody-Specific Inhibition
Source: PLoS One. 2012 Oct 29;7(10):e48172. doi: 10.1371/journal.pone.0048172 (PMC3483154; doi:10.1371/journal.pone.0048172)
Supplement: Table S1 — Thrombin generation parameters for MAbs at 10 µg/ml. ETP, peak thrombin and index velocity are presented as ratios compared to fVIII deficient plasma supplemented with 1 U/ml fVIII in the absence of any anti-fVIII MAb. ETP – endogenous thrombin potential. (DOC) [file pone.0048172.s001.doc]

Table S1

| **MAb** | **Thrombin Generation Parameter** | **[Mab] = 10µg/ml** | | | | | | | | | | | | | | | | | |
| --- | --- | --- | --- | --- | --- | --- | --- | --- | --- | --- | --- | --- | --- | --- | --- | --- | --- | --- | --- |
|  |  | **No Incubation** | | | | | | | | | **1 Hour Incubation** | | | | | | | | |
|  |  | **fVIII** | | | **rfVIIa** | | | **fVIII + fVIIa** | | | **fVIII** | | | **rfVIIa** | | | **fVIII + fVIIa** | | |
| None | ETP (ratio) | 1.0 | ± | 0.0 | 0.9 | ± | 0.0 | 1.9 | ± | 0.2 | 1.0 | ± | 0.1 | 0.9 | ± | 0.2 | 1.2 | ± | 0.6 |
|  | Peak (ratio) | 1.0 | ± | 0.0 | 0.4 | ± | 0.1 | 1.1 | ± | 0.1 | 1.0 | ± | 0.1 | 0.3 | ± | 0.1 | 1.2 | ± | 0.3 |
|  | Lag Time (min) | 5.6 | ± | 1.7 | 4.6 | ± | 1.3 | 4.1 | ± | 1.0 | 6.8 | ± | 2.1 | 6.3 | ± | 2.2 | 5.3 | ± | 1.6 |
|  | Peak Time (min) | 9.9 | ± | 1.9 | 11.9 | ± | 1.4 | 7.9 | ± | 1.4 | 12.5 | ± | 3.0 | 22.6 | ± | 14.0 | 10.0 | ± | 1.8 |
|  | Velocity (ratio) | 1.0 | ± | 0.0 | 0.4 | ± | 0.0 | 1.2 | ± | 0.4 | 1.0 | ± | 0.2 | 0.2 | ± | 0.1 | 1.4 | ± | 0.3 |
| 2-54 | ETP (ratio) | 1.4 | ± | 1.2 | 1.3 | ± | 0.7 | 1.7 | ± | 0.6 | 1.3 | ± | 0.4 | 1.2 | ± | 0.4 | 1.1 | ± | 0.4 |
|  | Peak (ratio) | 1.0 | ± | 0.2 | 0.4 | ± | 0.0 | 1.0 | ± | 0.1 | 1.2 | ± | 0.1 | 0.6 | ± | 0.2 | 1.1 | ± | 0.2 |
|  | Lag Time (min) | 5.8 | ± | 1.6 | 4.1 | ± | 1.0 | 4.4 | ± | 0.6 | 6.0 | ± | 2.4 | 5.1 | ± | 1.3 | 4.5 | ± | 1.3 |
|  | Peak Time (min) | 11.6 | ± | 3.5 | 11.9 | ± | 3.6 | 9.6 | ± | 2.6 | 14.6 | ± | 4.4 | 18.1 | ± | 6.4 | 11.3 | ± | 2.8 |
|  | Velocity (ratio) | 0.8 | ± | 0.2 | 0.4 | ± | 0.2 | 0.9 | ± | 0.3 | 0.7 | ± | 0.2 | 0.3 | ± | 0.3 | 0.9 | ± | 0.2 |
| G38 | ETP (ratio) | 1.5 | ± | 0.1 | 1.5 | ± | 0.3 | 1.7 | ± | 0.4 | 0.9 | ± | 0.4 | 1.0 | ± | 0.4 | 1.3 | ± | 0.1 |
|  | Peak (ratio) | 0.5 | ± | 0.1 | 0.3 | ± | 0.1 | 0.7 | ± | 0.0 | 0.4 | ± | 0.2 | 0.3 | ± | 0.1 | 0.8 | ± | 0.2 |
|  | Lag Time (min) | 6.4 | ± | 1.9 | 4.4 | ± | 0.6 | 4.4 | ± | 0.6 | 10.3 | ± | 6.3 | 6.7 | ± | 2.7 | 6.5 | ± | 1.6 |
|  | Peak Time (min) | 14.6 | ± | 4.4 | 13.8 | ± | 3.8 | 10.8 | ± | 2.0 | 22.2 | ± | 8.0 | 20.0 | ± | 7.7 | 16.6 | ± | 3.4 |
|  | Velocity (ratio) | 0.4 | ± | 0.2 | 0.3 | ± | 0.1 | 0.6 | ± | 0.1 | 0.2 | ± | 0.1 | 0.2 | ± | 0.1 | 0.4 | ± | 0.2 |
| 2-A9 | ETP (ratio) | 0.8 | ± | 1.5 | 0.6 | ± | 0.1 | 1.7 | ± | 0.2 | 1.1 | ± | 0.2 | 1.0 | ± | 0.7 | 1.2 | ± | 0.2 |
|  | Peak (ratio) | 0.8 | ± | 0.2 | 0.5 | ± | 0.0 | 0.9 | ± | 0.2 | 0.8 | ± | 0.1 | 0.4 | ± | 0.2 | 1.0 | ± | 0.1 |
|  | Lag Time (min) | 4.9 | ± | 1.0 | 3.6 | ± | 0.9 | 4.3 | ± | 0.8 | 7.0 | ± | 2.9 | 5.3 | ± | 2.5 | 5.0 | ± | 1.2 |
|  | Peak Time (min) | 10.8 | ± | 2.9 | 10.4 | ± | 2.4 | 8.3 | ± | 2.0 | 16.5 | ± | 3.8 | 23.0 | ± | 13.4 | 11.8 | ± | 2.4 |
|  | Velocity (ratio) | 0.7 | ± | 0.3 | 0.4 | ± | 0.1 | 1.1 | ± | 0.3 | 0.5 | ± | 0.2 | 0.2 | ± | 0.1 | 0.8 | ± | 0.1 |
| I109 | ETP (ratio) | 1.3 | ± | 0.7 | 0.9 | ± | 0.2 | 0.1 | ± | 0.4 | 0.8 | ± | 0.2 | 0.8 | ± | 0.2 | 0.8 | ± | 0.2 |
|  | Peak (ratio) | 0.5 | ± | 0.0 | 0.3 | ± | 0.0 | 0.6 | ± | 0.1 | 0.4 | ± | 0.1 | 0.3 | ± | 0.1 | 0.5 | ± | 0.1 |
|  | Lag Time (min) | 6.3 | ± | 1.6 | 4.8 | ± | 1.6 | 4.8 | ± | 1.2 | 7.3 | ± | 4.2 | 7.1 | ± | 1.9 | 6.6 | ± | 1.8 |
|  | Peak Time (min) | 14.3 | ± | 2.9 | 14.1 | ± | 4.0 | 11.1 | ± | 3.0 | 23.2 | ± | 5.2 | 21.3 | ± | 7.3 | 15.6 | ± | 3.5 |
|  | Velocity (ratio) | 0.4 | ± | 0.1 | 0.3 | ± | 0.1 | 0.5 | ± | 0.1 | 0.1 | ± | 0.1 | 0.1 | ± | 0.1 | 0.3 | ± | 0.1 |
| 2-77 | ETP (ratio) | 1.0 | ± | 0.2 | 1.4 | ± | 0.3 | 1.7 | ± | 0.4 | 0.9 | ± | 0.2 | 1.1 | ± | 0.3 | 1.0 | ± | 0.2 |
|  | Peak (ratio) | 0.5 | ± | 0.1 | 0.3 | ± | 0.0 | 0.7 | ± | 0.0 | 0.6 | ± | 0.2 | 0.4 | ± | 0.1 | 0.8 | ± | 0.1 |
|  | Lag Time (min) | 6.1 | ± | 2.2 | 4.6 | ± | 0.9 | 4.6 | ± | 1.3 | 8.5 | ± | 3.3 | 6.7 | ± | 1.4 | 6.0 | ± | 1.6 |
|  | Peak Time (min) | 14.1 | ± | 2.6 | 13.8 | ± | 4.3 | 10.6 | ± | 2.6 | 18.8 | ± | 3.3 | 17.8 | ± | 4.7 | 13.5 | ± | 2.9 |
|  | Velocity (ratio) | 0.4 | ± | 0.0 | 0.3 | ± | 0.1 | 0.6 | ± | 0.2 | 0.3 | ± | 0.2 | 0.2 | ± | 0.1 | 0.6 | ± | 0.2 |
